# Acceptability and feasibility of chemoprophylaxis with single-dose rifampicin in four leprosy-endemic districts in Benin

**DOI:** 10.1371/journal.pntd.0013057

**Published:** 2025-04-28

**Authors:** Parfait Djossou, Zinsou Franck Maurille Mignanwande, Sèdjro Gimatal Esaï Anagonou, Irene Cerda, Alice Toussaint, Jean Gabin Houezo, Ghislain Emmanuel Sopoh, Anna Gine March, Roch Christian Johnson

**Affiliations:** 1 Centre Interfacultaire de Formation et de Recherche en Environnement pour le Développement Durable, Université d’Abomey-Calavi, Cotonou, Bénin; 2 Fondation Anesvad, Bilbao, Espagne; 3 Ministry of Health, Programme National de Lutte contre la Lèpre et l’Ulcère de Buruli, Cotonou, Bénin; 4 Institut Régional de Santé Publique de Ouidah, Université d’Abomey-Calavi, Cotonou, Bénin; 5 Fondation Raoul Follereau, Paris, France; Instituto Butantan, BRAZIL

## Abstract

**Backgrounds:**

Chemoprophylaxis with single-dose rifampicin (SDR) is a preventive measure recommended by the World Health Organization to limit leprosy transmission. This study was carried out to assess the acceptability and feasibility of this measure in Benin.

**Methods:**

This intervention-oriented study, including contacts of people affected by leprosy (PALs), was conducted in two linear phases from September 2019 to August 2020 in Benin. In the first phase, we assessed contacts’ knowledge of leprosy and their perceptions of SDR through interviews conducted after their informed consent. In the second phase, contacts were educated about leprosy and the importance of SDR in leprosy control. Eligible contacts were clinically examined, and new leprosy patients were treated with multidrug therapy while consented healthy contacts received the SDR.

**Results:**

9,941 contacts were registered around 197 PALs. After interviewing 394 contacts, the majority (88.8%) had insufficient leprosy knowledge. Of these contacts, 58.6% agreed to receive chemoprophylaxis. They were willing to take the necessary time for treatment (74.5%), travel long distances (83.1%) and take the drug as often as possible (90.0%). Marital status (p = 0.008), education level (p = 0.000) and knowledge of leprosy (p = 0.000) were statistically linked to chemoprophylaxis acceptance. Contacts who lived alone, who weren’t educated and had little knowledge of leprosy were respectively 2.18, 2.75 and 43.13 times more likely to refuse chemoprophylaxis.

Of the 9,941 sensitized contacts, 6,798 were clinically examined, and 6,416 received SDR. The average age of contacts who received chemoprophylaxis was 27.3 years (±19.8), with a predominance of women (52.7%). Eight (8) new patients were identified and treated with multidrug therapy.

**Conclusion:**

It is important to increase awareness to improve knowledge of leprosy to contribute to chemoprophylaxis acceptance.

## Author summary

Despite the decline in leprosy cases in recent years, the incidence rate has stagnated, indicating that the transmission has not yet been broken. New approaches to reducing the number of cases and curbing disease transmission, such as early case detection, clinical contact examination and chemoprophylaxis, are now being recommended. In leprosy control, rifampicin chemoprophylaxis for contacts of patients is a preventive intervention that can prevent the transmission of infection among household members. We report here the experience of an intervention-oriented study in four leprosy-endemic localities in Benin. We first assessed the leprosy contacts knowledge of the disease, the acceptability and feasibility of single-dose rifampicin (SDR) before administering it to those who gave informed consent. Of these contacts, 58.6% agreed to receive chemoprophylaxis and were willing to take the necessary time for treatment and travel long distances to take the drug as often as possible. However, contacts who lived alone who weren’t educated and had little knowledge of leprosy were more likely to refuse chemoprophylaxis with SDR. It is therefore important to improve education and knowledge of the disease to contribute to the acceptance of chemoprophylaxis.

## Introduction

Leprosy, like other neglected tropical diseases, is one of the oldest diseases and is most prevalent among people living in poor communities **[1]**.

Its transmission is favored by promiscuity and close, prolonged skin contact with untreated patients **[2–4]**. However, close and prolonged contact with an untreated leprosy patient is a major risk factor **[5–7]**.

Despite the decrease in the number of leprosy cases detected, thanks to the introduction of effective treatment since the early 1980s, it is not possible to reduce incidence to zero and eliminate the source of infection **[8]**. Therefore, new approaches to reducing the number of cases and curbing disease transmission, such as early case detection, clinical contact examination and chemoprophylaxis, are now recommended **[9]**.

In leprosy control, rifampicin chemoprophylaxis for contacts of patients is a preventive intervention that can prevent the transmission of infection among household members **[10]**. Numerous randomized controlled trials have shown that postexposure chemoprophylaxis (PEP) reduces leprosy transmission, although its efficacy is controversial **[11–14]**. The effectiveness of PEP has been shown in Brazil, India, Indonesia, Burma, Cambodia, Bangladesh, Sri Lanka, Myanmar, Tanzania and Morocco **[**8,15–20**]**.

In Benin, leprosy remains a public health problem as its transmission persists in endemic communities despite the ongoing efforts of the country’s health authorities. Thus, 150 to 200 new leprosy cases are detected each year in Benin **[21]**. In 2022, national statistics also showed that the proportion of leprosy cases with grade 2 disability among new cases detected was 54%, with multibacillary (MB) cases accounting for 97%. These data highlight the public health problem that leprosy remains, despite the reduction in the number of cases **[22]**. To limit leprosy transmission, the prevention strategy of chemoprophylaxis with single-dose rifampicin (SDR) should be implemented in these endemic communities, following the examples of other leprosy-endemic countries **[**8,15–20**]**. The present study was therefore conducted to assess the acceptability and feasibility of chemoprophylaxis with SDR in the communities of Djidja, Ouinhi, Zagnanado and Kétou in Benin.

## Methods

### Ethics statement

The National Health Research Ethics Committee (CNERS) of the Benin of Ministry of Health reviewed the study proposal and approved the study on April 4, 2019 (No. 21/MS/DC/SGM/DRFMT/CNERS/SA/). Ethical considerations were respected during data collection. All participants who met the inclusion criteria were informed of the study’s objectives, procedures, advantages and disadvantages. The written and oral informed consent was obtained from all participants included in this study. From the parent/guardian of each participant under 18 years of age, written and oral informed consent was obtained.

### Setting

The study was carried out in four municipalities in Benin: Djidja, Ouinhi, Zagnanado (Zou Department) and Kétou (Plateau Department). These communities and the 96 villages investigated, which have different sociosanitary characteristics, were selected based on their leprosy endemicity according to National Buruli Ulcer and Leprosy Control Program of Bénin (PNLLUB) data.

According to data from Benin’s National Institute of Statistics and Economic Analysis (INSAE), the populations of Djidja, Ouinhi, Zagnanado and Kétou were estimated to be 146,681, 70,507, 65,377 and 186,834 inhabitants, respectively **[23]**.

### Study design and population

This intervention-oriented study, conducted from September 2019 to August 2020, was implemented in two (02) phases and included contacts of people affected by leprosy. The first phase assessed contacts’ knowledge of leprosy and perceptions of SDR. The second phase involved raising contacts’ awareness of the importance of SDR, followed by its administration.

The overall study population consisted of intra and extra domestic contacts of all persons affected by leprosy (PALs) registered over the period 2006-2016 in the study area. These contacts were family members of PALs and residents of neighbouring houses.

For the assessing knowledge of leprosy and perceptions of SDR, participants included were contacts aged 18 years old and over, available on the day of the survey and having given their consent.

For the awareness sessions, all contacts and PALs had participated. However, for the contact clinical examination and SDR administration, the participants included were:

- Family contacts living in the same households as the index patients (PALs);
- Direct neighbours whose plots touched the plots of the index patients;
- People living within a radius of no more than 150 m around the home of the PALs.

The participants not included were:

- Contacts who were absent on the day of the survey;
- Contacts who refused to participate in the study;
- Pregnant women;
- Children under 2 years old;
- People with reported leprosy who had been treated or were receiving treatment;
- People with tuberculosis and Buruli ulcer

### Sampling

Exhaustive sampling was used to select PALs from the period 2006–2016. Thus, 197 PALs were identified in the communities of Djidja, Ouinhi, Zagnanado and Kétou after an active search based on information provided by PNLLUB database. According to their proximity to the PALs, 9,941 contacts were registered.

For the assessment phase of leprosy knowledge and perceptions of SDR, the sample size of contacts interviewed around PALs was determined using the software Epi Info version 7.2.2.2. As the proportion of patients accepting chemoprophylaxis was not known in the localities covered by the study and given that no study of chemoprophylaxis had been carried out, we estimated a value of 50%. Assuming an acceptable margin of error of 5%, the sample size of contact was 384. For compliance reasons (assuming there will be non-responses and participants who would not be found), 394 contacts were selected. Contacts were chosen based on their proximity to a patient, i.e., a member of the patient’s household or compound or a close neighbour.

For the awareness and SDR administration phase, a rational selection was made of the contacts clinically examined and who received the SDR, according to the inclusion criteria.

### Variables

The dependent variable was acceptance of the use of rifampicin for chemoprophylaxis and the independent variables included sociodemographic data such as age, sex, education level, occupation and level of knowledge of the contacts about leprosy.

### Data collection

#### Active index case finding (PAL).

Using the PNLLUB database and community volunteers, health workers (leprosy nurse supervisors, state nurses, care assistants), local authorities (village chiefs and councillors), PALs detected between 2006 and 2016 were searched.

#### Registering of PALs contacts.

Once a PAL had been found, a study was explained to him/her and his entourage. People (contacts) within a radius of 150 meters of the index case who had given their consent were registered.

### Interviewing contacts about leprosy and acceptability of SDR

#### Informing participants and obtaining consent.

Eligible participants were verbally informed about the purpose of the study, its various stages and its benefits for the communities. The written and oral informed consent was obtained from all participants included in this study. From the parent/guardian of each participant under 18 years of age, written and oral informed consent was obtained. They were then invited to participate.

#### Contact interviews.

Among the contacts selected and registered according to inclusion criteria, the sampling was interviewed using a semi-structured questionnaire. Two contacts were interviewed for each leprosy patient.

### Awareness and administration of rifampicin chemoprophylaxis

#### Contacts’ awareness about leprosy and chemoprophylaxis.

Following the first phase, and after a reminder of the purpose, the various stages and the benefits of the study for the communities, awareness-raising was carried out in each intervention village, using the tools based on the results of the contact interviews (points of attention for community awareness-raising).

Contacts were informed of the next procedures of the study and possible outcomes (confirmed leprosy, no leprosy, eligible contact for SDR) of the different clinical examination methods. They were informed not only of the benefits of chemoprophylaxis with SDR such as reduction in the probability of contracting leprosy but also of its common side effects such as urine discolouration as well as less frequent or extremely rare adverse effects.

All eligible contacts were invited to participate in the next stages of the study (clinical contact examination, administration of SDR) after their consent. For the participant under 18 years old, the parent/guardian written and oral informed consent was obtained.

#### Clinical examination of contacts and administration of chemoprophylaxis with rifampicin.

Once consent has been obtained, contacts were clinically examined by leprosy specialists who looked for leprosy signs. Detected cases of leprosy were categorized according to the WHO classification **[24–26]**. Consenting healthy contacts received a supervised dose of rifampicin according to their age and weight. The dosage was applied according to WHO recommendations **[27]**. New leprosy patients were systematically treated with multidrug therapy.

### Data processing and analysis

The data collected were entered and analysed using SPSS 25 software. Frequencies and proportions were calculated for the qualitative variables. For quantitative variables, the means and standard deviations were determined. To assess the level of knowledge of PAL contacts, questions were formulated on a questionnaire. For each correct answer given, a score was assigned, and the maximum total score possible for an interviewed contact was ten (10). The total score obtained was used to classify the contact interviewed in one of 2 categories:

- Bad knowledge if the score obtained is < 5;
- Good knowledge if the score obtained is ≥ 5.

These scores were proposed and validated by leprosy specialists based on the relevance and importance of the appropriate answers to each question.

### Univariate analysis

The proportions of the dependent variable (acceptance of chemoprophylaxis) were compared with those of the various independent variables. Association between the different variables were investigated using the chi-square test and Fisher’s exact test. The significance threshold was 5%.

### Multivariate analysis

Variables with a P-value less than or equal to 0.2 were selected for multivariable analysis. Logistic regression was used with a progressive top-down elimination process to identify variables significantly associated with acceptance of chemoprophylaxis. P-values of 0.05 were considered statistically significant. The adequacy of the final model was investigated using the Hosmer-Lemeshow test.

## Results

### PALs identified and contacts registered

In this study, 197 PALs (index cases) were identified: 90 in Djidja, 40 in Ouinhi, 30 in Zagnanado and 37 in Kétou. Among these patients, 132 (67.0%) were multibacillary (MB) and 65 (33%) paucibacillary (PB).

Around the 197 PALs, 9,941 contacts were registered for the subsequent stages of the study.

### Knowledge of leprosy and perceptions of chemoprophylaxis

#### Sociodemographic characteristics of the respondents.

Of the 9,941 contacts registered around the 197 PALs, 394 were interviewed about leprosy and acceptability of chemoprophylaxis. The average age of contacts was 38.0 years (±13.4). Most contacts were married (81.2%), male (50.5%), and Fons ethnic group (78.2%), had not attended school (50.5%), practiced Christianity (68.3%), were cultivators (50.5%), housekeepers (15.0%), students (11.4%) and retailers (8.4%) (Table 1).

**Table 1:** Sociodemographic characteristics of the contacts interviewed about knowledge of leprosy and perception of chemoprophylaxis.

Features	Djidja n (%)	Ouinhi n (%)	Zagnanado n (%)	Kétou n (%)	Total n (%)
Age*					
Average (Standard deviation)	39.4 (+/-12.1)	34.7 (+/-14.0)	38.6 (+/-18.1)	37.8 (+/-10.9)	38 (+/-13.4)
**Sex**					
Male	99 (55.0)	35 (43.8)	29 (48.3)	36 (48.6)	199 (50.5)
Female	81 (45.0)	45 (56.2)	31 (51.7)	38 (51.4)	195 (49.5)
**Marital status**					
Single	14 (7.7)	24 (30.0)	18 (30.0)	5 (6.8)	61 (15.5)
Married	164 (91.1)	55 (68.7)	32 (53.3)	69 (93.2)	320 (81.2)
Divorced	1 (0.6)	0 (0.0)	6 (10.0)	0 (0.0)	7 (1.8)
Widowed	1 (0.6)	1 (1.3)	4 (6.7)	0 (0.0)	6 (1.5)
**Sociocultural group**					
Fon	172 (95.6)	76 (95.0)	60 (100.0)	0 (0.0)	308 (78.2)
Holli	0 (0.0)	4 (5.0)	0 (0.0)	64 (86.4)	68 (17.4)
Yoruba	0 (0.0)	0 (0.0)	0 (0.0)	5 (6.8)	5 (1.3)
Nago	0 (0.0)	0 (0.0)	0 (0.0)	5 (6.8)	5 (1.3)
Other**	8 (4.4)	0 (0.0)	0 (0.0)	0 (0.0)	8 (2.0)
**Religion**					
Christianity	138 (76.7)	69 (86.2)	43 (71.7)	19 (25.7)	269 (68.3)
Muslim	12 (6.7)	4 (5.0)	3 (5.0)	22 (29.7)	41 (10.4)
Traditional	30 (16.7)	7 (8.8)	14 (23.3)	33 (44.6)	84 (21.3)
**School level**					
None	95 (52.8)	41 (51.3)	23 (38.4)	40 (54.1)	199 (50.5)
Primary	72 (40.0)	23 (28.8)	21 (35.0)	25 (33.8)	141 (35.8)
Secondary 1	7 (3.9)	11 (13.7)	14 (23.3)	5 (6.7)	37 (9.4)
Secondary 2	6 (3.3)	5 (6.2)	2 (3.3)	4 (5.4)	17 (4.3)
**Profession**					
Retailer	5 (2.8)	5 (6.2)	3 (5.0)	20 (27.0)	33 (8.4)
Housekeeper	29 (16.1)	16 (20.0)	6 (10.0)	8 (10.8)	59 (15.0)
Student	18 (10.0)	15 (18.8)	10 (16.7)	2 (2.7)	45 (11.4)
Civil servant	2 (1.1)	1 (1.3)	0 (0.0)	3 (4.1)	6 (1.5)
Apprentice***	4 (2.2)	9 (11.3)	7 (11.7)	1 (1.4)	21 (5.3)
Cultivator	118 (65.6)	26 (32.5)	23 (38.3)	32 (43.2)	199 (50.5)
Fisherman	0 (0.0)	3 (3.7)	10 (16.7)	0 (0.0)	13 (3.3)
Artisan	4 (2.2)	5 (6.2)	1 (1.6)	8 (10.8)	18 (4.6)

*For age, the average and standard deviation are calculated; **Other: Adja (n = 3), Agoun (n = 5); ***Apprentice: hairdressing, sewing

#### Basic knowledge of the contacts of PALs.

Among the 394 people interviewed in the four communities, the majority (74.9%) recognized leprosy most often because of the presence of visible deformities. Almost all of these people (99.2%) knew nothing about the signs of leprosy onset. Few contacts (39.8%) knew the real cause of leprosy (a microorganism). The contagiousness of leprosy was known by 89.8% of the contacts, and 17.8% knew the mode of transmission (human-to-human). Most contacts thought that leprosy was curable (97.2%) and to treat it, they had to go to hospital (86.5%) (Table 2).

**Table 2:** Contacts’ knowledge of leprosy in the communities of Djidja, Ouinhi, Zagnanado and Kétou.

Features	Djidja n (%)	Ouinhi n (%)	Zagnanado n (%)	Kétou n (%)	Total n (%)
**Knew the signs of leprosy**					
Yes	141 (78.3)	61 (76.2)	46 (76.7)	47 (63.5)	295 (74.9)
No	39 (21.7)	19 (23.8)	14 (23.3)	27 (36.5)	99 (25.1)
**Knew the early signs of leprosy**					
Yes	2 (1.1)	1 (1.3)	0 (0.0)	0 (0.0)	3 (0.8)
No	178 (98.9)	79 (98.7)	60 (0.0)	74 (100.0)	391 (99.2)
**Knew the cause of leprosy**					
Yes	77 (42.8)	31 (38.8)	23 (38.3)	26 (35.1)	157 (39.8)
No	103 (57.2)	49 (61.2)	37 (61.7)	38 (64.9)	237 (60.2)
**Knew the contagiousness of leprosy**					
Yes	162 (90.0)	71 (88.8)	54 (90.0)	67 (90.5)	354 (89.8)
No	18 (10.0)	9 (11.2)	6 (10.0)	7 (9.5)	40 (10.2)
**Knew the transmission mode**					
Yes	41 (22.8)	13 (16.3)	7 (11.7)	9 (12.2)	70 (17.8)
No	139 (77.2)	67 (83.7)	53 (88.3)	48 (87.8)	324 (82.2)
**Knew that leprosy can be cured**					
Yes	176 (97.8)	78 (97.5)	58 (96.7)	71 (96.0)	383 (97.2)
No	4 (2.2)	2 (2.5)	2 (3.3)	3 (4.0)	11 (2.8)
**Knew that leprosy can be treated in the hospital**					
Yes	159 (88.3)	67 (83.8)	52 (86.7)	63 (85.1)	341 (86.5)
No	21 (11.7)	13 (16.2)	8 (13.3)	11 (14.9)	53 (13.5)
					

#### Perceptions of chemoprophylaxis among the contacts of PALs.

**Fig 1:**
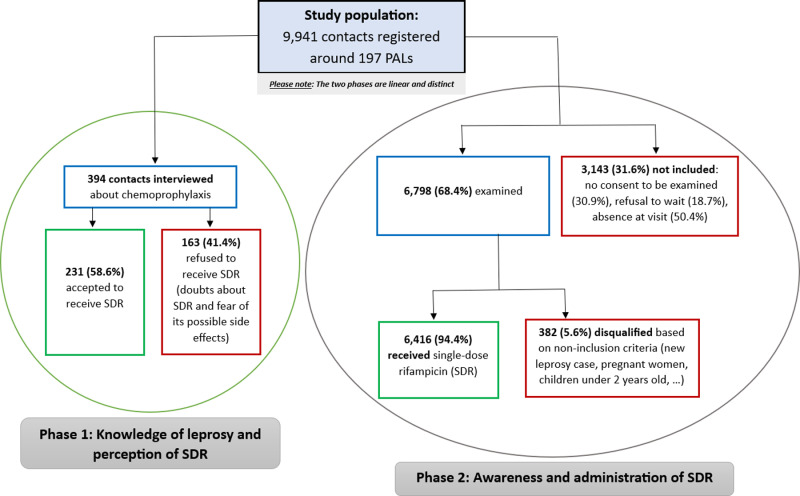
Study organization diagram with the main results.

Of the 394 contacts interviewed about chemoprophylaxis with rifampicin in the communities of Djidja, Ouinhi, Zagnanado and Kétou, 58.6% wanted to receive it (Fig 1). Of the contacts who agreed to receive chemoprophylaxis with rifampicin, the majority were willing to take the drug in any form (73.6%). These contacts were willing to take the necessary time for treatment (74.5%) and travel long distances (83.1%). They were also willing to take the drug as many times as required (90.0%), even if it had undesirable side effects (83.5%) (Table 3).

**Table 3:** Contacts’ perceptions of chemoprophylaxis.

Variables/Terms and conditions	Djidja n (%)	Ouinhi n (%)	Zagnanado n (%)	Kétou n (%)	Total n (%)
**Acceptance of chemoprophylaxis**					
Yes	139 (77.2)	33 (41.3)	20 (33.3)	39 (52.7)	231 (58.6)
No	41 (22.8)	47 (58.7)	40 (66.7)	35 (47.3)	163 (41.4)
**Preferred forms of medication**					
Tablet	29 (20.9)	12 (36.4)	8 (40.0)	6 (15.4)	55 (23.8)
Syrup	3 (2.2)	7 (21.2)	4 (20.0)	3 (7.7)	17 (7.4)
Capsule	8 (5.8)	7 (21.2)	3 (15.0)	1 (2.6)	19 (8.2)
Injection	2 (1.4)	1 (3.0)	0 (0.0)	0 (0.0)	3 (1.3)
Any form	108 (77.7)	19 (57.6)	11 (55.0)	32 (82.0)	170 (73.6)
**Willing to travel long distances to receive chemoprophylaxis**					
Yes	118 (84.9)	28 (84.8)	15 (75.0)	31 (79.5)	192 (83.1)
No	21 (15.1)	5 (15.2)	5 (25.0)	8 (20.5)	39 (16.9)
**Willing to spend even a whole day receiving chemoprophylaxis**					
Yes	106 (76.3)	28 (84.8)	11 (55.0)	27 (69.2)	172 (74.5)
No	33 (23.7)	5 (15.2)	9 (45.0)	12 (30.8)	59 (25.5)
**Number of times chemoprophylaxis can be administered**					
Once	8 (5.8)	2 (6.1)	0 (0.0)	4 (10.3)	14 (6.1)
Two (2) times	3 (2.1)	1 (3.0)	4 (20.0)	1 (2.5)	9 (3.9)
As many times	128 (92.1)	30 (90.9)	16 (80.0)	34 (87.2)	208 (90.0)
**Acceptance of chemoprophylaxis despite side effects**					
Yes	113 (81.3)	29 (87.9)	18 (90.0)	33 (84.6)	193 (83.5)
No	26 (18.7)	4 (12.1)	2 (10.0)	6 (15.4)	38 (16.5)

The results showed that the majority of those questioned (58.6%) were in favour of chemoprophylaxis against leprosy. This was also reflected in the opinions gathered, such as that of this septuagenarian from the village of Fonkpodji in Djidja, who said: “*We have been waiting a very long time for a drug that can prevent leprosy, and now it is finally here. I will take it several times according to your recommendations if it can protect me and my family from this humiliating disease that affects so many people in our region*.” Indeed, this father of a family has recognized the need to prevent the disease in himself and his family to avoid the humiliation that this pathology entails.

For others who accepted chemoprophylaxis, the cost of this preventive intervention remained a problem for the population. This highlights the economic limitations of these populations, who often have a modest income but want to preserve their health whatever the possibility. In Agonvè, in the municipality of Zagnanado, a woman in her fifties said: “*This medicine that you are offering us free of charge will protect us and our families from this disease that causes so much damage to its victims. We accept it with open arms because it is for our own good*”.

Although most individuals were willing to receive chemoprophylaxis, some people did not accept the idea, even if the intervention was free of charge. The reasons given were generally doubts about the drug administered and fears of possible side effects. In Kétou, in a locality made up of the Holli ethnic group, the community refused chemoprophylaxis. One individual stated: “*You give us this kind of medication and then we see health problems such as loss of sight, which many of us are currently experiencing without any solution following the mass distribution of a medication in the past. We do not want to make our situation worse, so we do not want to receive your medication*”. This refusal is then motivated by past events not related to the present study and which are said to have caused irreversible health damage in the community. Awareness-raising sessions such as behaviour change communication could change their view on chemoprophylaxis. In the community of Zagnanado, a divorced woman who refused chemoprophylaxis said: “*Personally, I don’t trust your medicine because I have never heard of it before. So, I prefer not to take it*.” Here again, it is possible to enable the populations to better understand and control the implications of chemoprophylaxis by organizing awareness-raising and study popularization sessions.

The multivariate analyses showed that variables such as marital status (p=0.008), education level (p=0.000) and knowledge of leprosy (p=0.000) were significantly related to the contacts’ acceptance of chemoprophylaxis with rifampicin. However, contacts who lived alone (single, widowed, or divorced), those with no education and those with poor knowledge of leprosy were 2.18, 2.75 and 43.13 times more likely to refuse chemoprophylaxis with rifampicin, respectively (Tables 4 and 5).

**Table 4:** Results of univariate analysis of the receipt of chemoprophylaxis by PALs contacts.

	Receipt of chemoprophylaxis				
**Variables/Terms and conditions**	**No n (%)**	**Yes n (%)**	**Odds Ratio**	**95% CI**	**p value**
**Age**					
< 18 years	18 (11.0)	5 (2.2)	1	–	
[18-25 years]	17 (10.4)	37 (16.0)	0.12	0.04-0.40	0.001*
[26-40 years]	59 (36.2)	103 (44.6)	0.15	0.05-0.45	
[41-60 years]	55 (33.7)	74 (32.0)	0.2	0.07-0.59	
> 60 years	14 (8.6)	12 (5.2)	0.32	0.09-1.13	
**Sex**					
Male	72 (44.2)	127 (55.0)	0.8	0.65-0.99	0.035*
Female	91 (55.8)	104 (45.0)	1.24	1.01-1.51	
**Marital status**					
Single/Divorced/	36 (22.1)	38 (16.5)	1.34	0.89-2.02	0.158
Widowed					
Married	127 (77.9)	193 (83.5)	0.93	0.84-1.03	
**Sociocultural group**					
Fon and related groups	128 (78.5)	188 (81.4)	0.96	0.87-1.06	0.483
Holli and related groups	35 (21.5)	43 (18.6)	1.15	0.77-1.71	
**Religion**					
Christian/Muslim	127 (77.9)	183 (79.2)	0.98	0.88-1.09	0.755
Traditional	36 (22.1)	48 (20.8)	1.06	0.72-1.55	
**Level of education**					
No education	102 (62.6)	97 (42.0)	1.49	1.22-1.80	0.000*
Educated	61 (37.4)	134 (58.0)	0.64	0.51-0.80	
**Profession**					
Farmer/Fisherman	86 (52.8)	125 (54.1)	0.97	0.80-1.17	
Other	77 (47.2)	106 (45.9)	1.02	0.83-1.27	0.791
**Knowledge about leprosy**					
Bad	162 (99.4)	188 (81.4)	1.22	1.14-1.30	0.000^*^
Good	1 (0.6)	43 (18.6)	0.03	0.00-0.23	

*p value significant according to Pearson’s Chi-square test

**Table 5:** Multivariate model of factors associated with the receipt of chemoprophylaxis with rifampicin.

Variables	Adjusted OR	95% CI	p value
Age	1	0.77-1.31	0.949
Sex	0.76	0.49-1.17	0.219
Marital status	2.18	1.22-3.89	0.008^*^
Level of education	2.75	1.75-4.33	0.000^*^
Knowledge about leprosy	43.13	5.77-321.98	0.000^*^

*p value significant according to Hosmer-Lemeshow test (logistic regression)

### Implementation of chemoprophylaxis in the communities of Djidja, Ouinhi, Zagnanado and Kétou

#### Clinical examination of contacts and chemoprophylaxis administration.

Following the leprosy and chemoprophylaxis awareness sessions, 6,798 contacts (68.4%) were clinically examined out of the 9,941 contacts recorded. 6,416 (94.4%) received rifampicin chemoprophylaxis and 382 (5.6%) did not (Fig 1). 2,472 contacts received chemoprophylaxis around 90 PALs in Djidja; 1,579 contacts received chemoprophylaxis around 40 PALs in Ouinhi; 1,564 contacts received chemoprophylaxis around 30 PALs in Zagnanado and 801 contacts received chemoprophylaxis around 37 PALs in Kétou. The average age of contacts who received chemoprophylaxis was 27.3 years (±20.3), with a predominance of females (52.7%) (Table 6).

**Table 6:** Sociodemographic characteristics of contacts clinically examined and received chemoprophylaxis.

Features	Djidja (n = 2,472)	Ouinhi (n = 1,579)	Zagnanado (n = 1,564)	Kétou (n = 801)	Total (n = 6,416)
**Age**					
Average (standard deviation)	23.7 (+/-19,8)	30.5 (+/-19,6)	31.6 (+/-21,1)	23.6 (+/-18,7)	27.3 (+/-20.3)
**Sex**					
Male	1,151 (46,6%)	775 (49,1%)	709 (45,3%)	400 (49,9%)	3,035 (47,3%)
Female	1,321 (53,4%)	804 (50,9%)	855 (54,7%)	401 (50,1%)	3,381 (52,7%)

#### New cases of leprosy detected during chemoprophylaxis.

During clinical examination of contact, height (8) new cases of leprosy (4 MB and 4 PB) were detected. Four (04) (2 MB and 2 PB) were detected in Djidja, two (02) (1 MB and 1 PB) were detected in Ouinhi and two (02) (1 MB and 1 PB) were detected in Zagnanado.

## Discussion

The present study on chemoprophylaxis is the first on this strategy in Benin. It enrolled 9,941 contacts around 197 PALs in four communities where leprosy is endemic: Djidja, Ouinhi, Zagnanado and Kétou.

In the first phase of this study, devoted to assessing contacts’ knowledge of leprosy and their perceptions of the SDR, 394 people were interviewed. The participants were married men and women, the majority of whom were uneducated (50.5%) and Christian (68.3%). For the most part, these contacts had erroneous knowledge of the cause of leprosy, its mode of transmission and the signs of disease onset. This could lead some people to be unreceptive to chemoprophylaxis. This situation also highlights the need for health education in these communities. The same point was made by Rajkumar *et al*., who stipulated in their study that it would be necessary to promote knowledge of leprosy within the community by means of an educational intervention that would enable knowledge and methods of leprosy control to be discussed **[28]**.

Of the 394 contacts interviewed, 231 (58.6%) agreed to receive chemoprophylaxis, while 163 (41.4%) refused it. Of the contacts who agreed to receive chemoprophylaxis, the majority were willing to take the drug in any form (74.5%). These contacts were even willing to take the necessary time for treatment (74.5%) and travel long distances (83.1%). They were also willing to take the drug as often as necessary (90.0%), even if it had undesirable side effects (83.5%). These results show that chemoprophylaxis with SDR is socially accepted in the communities of Djidja, Ouinhi, Zagnanado and Kétou. Similar results were obtained by Ferreira *et al*. and Apte *et al*. who reported that clinical contact examination and the distribution of SDR were very well accepted by the main stakeholders: index patients, contacts, health workers and supervisors **[**10,29**]**. The same is true for Schoenmakers *et al*., who found that the implementation of contact tracing and the administration of SDR in different leprosy control programmes was shown to be feasible and well accepted **[30]**. In addition, Richardus *et al.* demonstrated the feasibility of SDR in seven endemic countries in Asia, Africa and South America **[15]**.

Of the 394 contacts interviewed in this study, 163 (41.4%) expressed a refusal to receive chemoprophylaxis. The reasons given by some contacts for refusing chemoprophylaxis were generally doubts about the drug administered and fear of its possible side effects. Cases of refusal of chemoprophylaxis were also reported in a study carried out in Cambodia by Cavaliero *et al*. **[18]**.

In our study, multivariate analysis revealed that marital status (p=0.008), education level (p=0.000) and knowledge of leprosy (p=0.000) were significantly related to the acceptance of chemoprophylaxis with rifampicin in the four communities. Indeed, contacts who were not living with a partner (single, widowed, or divorced), who had no education and who had poor knowledge of leprosy were 2.185, 2.759 and 43.134 times more likely to refuse chemoprophylaxis with rifampicin, respectively. These factors could be corrected by improving contacts’ level of knowledge about the disease. This could be achieved through local awareness campaigns (in hamlets or villages where cases of leprosy are detected). Once contacts’ knowledge of leprosy has improved, they will be able to understand and integrate the meaning and role of chemoprophylaxis with rifampicin. These results are in line with those obtained by Cortela *et al*., who found that the receipt of postexposure chemoprophylaxis (PEP) was influenced by understanding, acceptance and expectation of the intervention. According to the same study, understanding was related to the care of the health team. Acceptance or nonacceptance of the drug was linked to fear, confidence and protection, the operationality of the strategy, self-esteem and insecurity about the intervention.

Following the first phase of our study, which uncovered leprosy-related gaps in the communities, awareness sessions were organized, and 9,941 contacts were registered. Then, 6,416 contacts (94.4%) received the SDR and 382 (5.6%) were disqualified based on noninclusion criteria among the 6,798 contacts examined. The average age of the people who received chemoprophylaxis was 27.3 years (±20.3), with a predominance of women (52.7%). These results reflect the situation at the national level, which shows a predominance of females in the Beninese population according to the 4^th^ global population and housing census conducted by the National Institute of Statistics and Economic Analysis **[23]**.

Of the 9,941 contacts registered during our study, 3,143 (31.6%) were not clinically examined. Reasons included absence the day of examination (50.4%), lack of consent (30.9%) and refusal to wait (18.7%). Lack of consent reflects a lack of understanding and recognition of the importance of SDR, and therefore a lack of receptiveness to the intervention on the part of some people, despite the organization of awareness-raising sessions for them. It would be important to amplify these awareness-raising sessions to increase the chances of convincing these latecomers to adhere to the intervention. If there were a sufficient number of medical teams, waiting times could be reduced by receiving participants as early as possible. This would make it easier for the participants, who are mostly farmers, to benefit from the intervention and to be able to go about their farming activities.

This result is similar to that obtained by Cavaliero *et al.* in a study carried out in 2021 in Cambodia, where it was found that out of 10,410 identified contacts of 555 index patients, 72.0% were clinically examined, while most of the others were absent the day of examination **[31]**.

Overall, chemoprophylaxis with rifampicin was mostly accepted by the communities of Djidja, Ouinhi, Zagnanado and Kétou in Benin. The same overall observation was made by Richardus *et al.* (2021), who found that PEP with SDR was generally well accepted by index patients, their contacts and healthcare workers in their multicountry study **[15]**. However, it is important to increase awareness of leprosy and measures to prevent it to ensure greater acceptance of chemoprophylaxis with rifampicin for future interventions in at-risk communities in Benin.

Despite these results, our study has some limitations. The perceptions of health staff and leprosy patients were not presented in this study. In addition, the effectiveness of SDR was not addressed, and by extension, the effect on reducing leprosy transmission was not studied. Contact follow-up was not carried out in this study, given the relatively short study period. Indeed, the average incubation period of leprosy is relatively long (5 to 10 years), short-term contact monitoring was not feasible. Further studies will consider this aspect.

## Conclusion

This study showed that chemoprophylaxis with SDR is socially accepted and can be implemented in leprosy-endemic communities in Benin. Marital status, education level and knowledge of leprosy among contacts of PALs are factors influencing the acceptance of chemoprophylaxis.

The little knowledge of contacts about leprosy revealed by this study, especially about the cause, mode of transmission and clinical signs of the disease, calls not only for community awareness-raising interventions but also for knowledge, attitude and practice studies to be carried out to gain a better understanding of the situation. It would be desirable for this type of study to be carried out prior to any rifampicin chemoprophylaxis intervention in leprosy-endemic communities. This would guarantee a high level of acceptance of the strategy, thanks to educational sessions that would have already considered the realities of each community.

## Supporting information

S1 Appendix STROBE-checklist-v4-cross-sectional. (PDF)

S2 Appendix Questionnaire Assessment the level of knowledge of PAL contact. (PDF)

S3 Appendix Questionnaire for contacts. (PDF)

S4 Appendix Assessment of the level of knowledge of PAL contact (Score). (DOCX)

## References

[1] Pescarini JM, Strina A, Nery JS, Skalinski LM, Andrade KVF de, Penna MLF, et al. Socioeconomic risk markers of leprosy in high-burden countries: A systematic review and meta-analysis. PLoS Negl Trop Dis. 2018;12(7):e0006622. doi: 10.1371/journal.pntd.0006622 29985930 PMC6053250

[2] Rodrigues LC, Lockwood DN. Leprosy now: epidemiology, progress, challenges, and research gaps. Lancet Infect Dis. 2011;11(6):464–70. doi: 10.1016/S1473-3099(11)70006-8 21616456

[3] Bosch X. Fontilles faces the future of leprosy. Lancet Infect Dis. 2003;3(4):185. doi: 10.1016/s1473-3099(03)00599-1 12679256

[4] Ploemacher T, Faber WR, Menke H, Rutten V, Pieters T. Reservoirs and transmission routes of leprosy; A systematic review. PLoS Negl Trop Dis. 2020;14(4):e0008276. doi: 10.1371/journal.pntd.0008276 32339201 PMC7205316

[5] Moet FJ, Pahan D, Schuring RP, Oskam L, Richardus JH. Physical distance, genetic relationship, age, and leprosy classification are independent risk factors for leprosy in contacts of patients with leprosy. J Infect Dis. 2006;193(3):346–53. doi: 10.1086/499278 16388481

[6] Romero-Montoya M, Beltran-Alzate JC, Cardona-Castro N. Evaluation and monitoring of mycobacterium leprae transmission in household contacts of patients with Hansen’s Disease in Colombia. PLoS Negl Trop Dis. 2017;11(1):e0005325. doi: 10.1371/journal.pntd.0005325 28114411 PMC5289623

[7] Wang L, Wang H, Yan L, Yu M, Yang J, Li J, et al. Single-dose rifapentine in household contacts of patients with leprosy. N Engl J Med. 2023;388(20):1843–52. doi: 10.1056/NEJMoa2205487 37195940

[8] Khoudri I, Elyoussfi Z, Mourchid Y, Youbi M, Bennani Mechita N, Abouqal R, et al. Trend analysis of leprosy in Morocco between 2000 and 2017: evidence on the single dose rifampicin chemoprophylaxis. PLoS Negl Trop Dis. 2018;12(12):e0006910. doi: 10.1371/journal.pntd.0006910 30571740 PMC6301570

[9] World Health Organization. Guidelines for the diagnosis, treatment and prevention of leprosy: Report of the literature review. 2018.

[10] Ferreira SMB, Yonekura T, Ignotti E, Oliveira LB de, Takahashi J, Soares CB. Effectiveness of rifampicin chemoprophylaxis in preventing leprosy in patient contacts: a systematic review of quantitative and qualitative evidence. JBI Database System Rev Implement Rep. 2017;15(10):2555–84. doi: 10.11124/JBISRIR-2016-003301 29035966

[11] Moet FJ, Pahan D, Oskam L, Richardus JH, COLEP Study Group. Effectiveness of single dose rifampicin in preventing leprosy in close contacts of patients with newly diagnosed leprosy: cluster randomised controlled trial. BMJ. 2008;336(7647):761–4. doi: 10.1136/bmj.39500.885752.BE 18332051 PMC2287265

[12] Dos Santos DS, Duppre NC, Sarno EN, Pinheiro RO, Sales AM, Nery JADC, et al. Chemoprophylaxis of leprosy with rifampicin in contacts of multibacillary patients: study protocol for a randomized controlled trial. Trials. 2018;19(1):244. doi: 10.1186/s13063-018-2623-6 29685164 PMC5914061

[13] Smith WC. Chemoprophylaxis in the prevention of leprosy. BMJ. 2008 Apr 5;336(7647):730–1. doi: 10.1136/bmj.39525.504688.80 18390497 PMC2287231

[14] Mieras LF, Taal AT, van Brakel WH, Cambau E, Saunderson PR, Smith WCS, et al. An enhanced regimen as post-exposure chemoprophylaxis for leprosy: PEP+. BMC Infect Dis. 2018;18(1):506. doi: 10.1186/s12879-018-3402-4 30290790 PMC6173927

[15] Richardus JH, Tiwari A, Barth-Jaeggi T, Arif MA, Banstola NL, Baskota R, et al. Leprosy post-exposure prophylaxis with single-dose rifampicin (LPEP): an international feasibility programme. Lancet Glob Health. 2021;9(1):e81–90. doi: 10.1016/S2214-109X(20)30396-X 33129378

[16] Tiwari A, Dandel S, Djupuri R, Mieras L, Richardus JH. Population-wide administration of single dose rifampicin for leprosy prevention in isolated communities: a three year follow-up feasibility study in Indonesia. BMC Infect Dis. 2018;18(1):324. doi: 10.1186/s12879-018-3233-3 29996781 PMC6042242

[17] Idema WJ, Majer IM, Pahan D, Oskam L, Polinder S, Richardus JH. Cost-effectiveness of a chemoprophylactic intervention with single dose rifampicin in contacts of new leprosy patients. PLoS Negl Trop Dis. 2010;4(11):e874. doi: 10.1371/journal.pntd.0000874 21072235 PMC2970532

[18] Cavaliero A, Greter H, Fürst T, Lay S, Sao Ay S, Robijn J, et al. An innovative approach to screening and chemoprophylaxis among contacts of leprosy patients in low endemic settings: experiences from Cambodia. PLoS Negl Trop Dis. 2019;13(3):e0007039. doi: 10.1371/journal.pntd.0007039 30921325 PMC6438440

[19] Feenstra SG, Nahar Q, Pahan D, Oskam L, Richardus JH. Acceptability of chemoprophylaxis for household contacts of leprosy patients in Bangladesh: a qualitative study. Lepr Rev. 2011;82(2):178–87. doi: 10.47276/lr.82.2.178 21888142

[20] Cortela DDCB, Ferreira SMB, Virmond MCL, Mieras L, Steinmann P, Ignotti E, Cavaliero A. Aceitabilidade da quimioprofilaxia em área endêmica para a hanseníase: projeto PEP-Hans Brasil [Acceptability of chemoprophylaxis in an endemic area for leprosy: the PEP-Hans Brazil Project]. Cad Saude Publica. 2020 Apr 3;36(3):e00068719. Portuguese. doi: 10.1590/0102-311X00068719 32267374

[21] Programme National de Lutte contre la Lèpre et l’Ulcère de Buruli (PNLLUB). Base de données sur la lèpre par départements et par communes de 2006 à 2016 au Bénin. Cotonou/Bénin; 2016.

[22] Programme National de Lutte contre la Lèpre et l’Ulcère de Buruli (PNLLUB). Rapport statistique annuel de la lèpre et de l’ulcère de Buruli. Cotonou, Bénin: Ministère de la Santé; 2022.

[23] Institut National des Statistiques et de l’Analyse Economique (INSAE). Effectifs de la population des villages et Quartiers de ville du Bénin (RGPH4). 2016.

[24] Géniaux M. Lèpre ou pas lèpre? Bulletin de l’ALLF. 2010;25:26.

[25] Fédération Internationale des Associations contre la Lèpre. Guide pédagogique Un: Comment diagnostiquer et traiter la lèpre. Genève/Suisse: ILEP; 2001.

[26] Organisation Mondiale de la Santé. Stratégie mondiale de lutte contre la lèpre 2016-2020: « Parvenir plus rapidement à un monde exempt de lèpre ». Genève/Suisse: OMS; 2016.

[27] Organisation Mondiale de la Santé. La lèpre ou maladie de Hansen: recherche des contacts et prophylaxie post-exposition. Orientations techniques. 2020.

[28] Rajkumar E, Julious S, Salome A, Jennifer G, John AS, Kannan L, et al. Effects of environment and education on knowledge and attitude of nursing students towards leprosy. Indian J Lepr. 2011;83(1):37–43. 21638982

[29] Apte H, Chitale M, Das S, Manglani PR, Mieras LF. Acceptability of contact screening and single dose rifampicin as chemoprophylaxis for leprosy in Dadra and Nagar Haveli, India. Lepr Rev. 2019;90(1):31–45.

[30] Schoenmakers A, Mieras L, Budiawan T, van Brakel WH. The state of affairs in post-exposure leprosy prevention: a descriptive meta-analysis on immuno- and chemo-prophylaxis. Res Rep Trop Med. 2020;11:97–117. doi: 10.2147/RRTM.S190300 33117053 PMC7573302

[31] Cavaliero A, Ay SS, Aerts A, Lay S, So V, Robijn J, et al. Preventing leprosy with retrospective active case finding combined with single-dose rifampicin for contacts in a low endemic setting: results of the Leprosy Post-Exposure Prophylaxis program in Cambodia. Acta Trop. 2021;224:106138. doi: 10.1016/j.actatropica.2021.106138 34562427

