# Supplementary material for: Acceptability and feasibility of chemoprophylaxis with single-dose rifampicin in four leprosy-endemic districts in Benin
Source: PLoS Negl Trop Dis. 2025 Apr 28;19(4):e0013057. doi: 10.1371/journal.pntd.0013057 (PMC12058174; doi:10.1371/journal.pntd.0013057)
Supplement: S2 Appendix — (PDF) [file pntd.0013057.s002.pdf]

To assess the level of **knowledge** of PAL contacts, questions were formulated on a questionnaire. For each correct answer given, a score was assigned and the maximum possible total score that an interviewed contact can have is ten (10). The total score obtained made it possible to classify the interviewed contact in one of the 2 categories, namely:

- *Bad :* if the score obtained is < 5;
- *Good :* if the score obtained is ≥ 5.

Operational aspects of variables for the assessment of contacts' knowledge of leprosy

Variables	Variable type<br>Terms/Code	Max score	Terms
**Knew the signs of leprosy**	*Nominal qualitative variable (multiple choice with 1=Yes and 0=No for each modality)*<br>a) Visible deformation<br>b) Light insensitive spots<br>c) Big nerves<br>d) Don't know	2	Answers a and c each have a weight of 0.5. Answer b is rated 1. Their sum determines the score
**Knew the early signs of leprosy**	*Nominal qualitative variable (multiple choice with 1=Yes and 0=No)*<br>a) Visible deformation<br>b) Light insensitive spots<br>c) Big nerves<br>d) Don't know	2	The correct answers b and c each have a weight of 1. Their sum determines the score.
**Knew the causes of leprosy**	*Nominal qualitative variable (multiple choice with 1=Yes and 0=No)*<br>a) Bewitchment or bewitchment<br>b) Divine punishment<br>c) Natural cause<br>d) Microorganism<br>e) Don't know	1	The correct answer has a weight of 1 and thus determines the score.
**Knew the contagiousness of leprosy**	*Binary qualitative variable*<br>1=Yes<br>0=No	0.5	The correct answer has a weight of 0.5 and thus determines the score.
**Knew the transmission mode**	*Nominal qualitative variable (multiple choice with 1=Yes and 0=No for each modality)*<br>1=From parent to child<br>2=From a sick person to a healthy person<br>3=Not transmitted<br>4=Don't know	1	The correct answer has a weight of 1 and thus determines the score.

**Knew that leprosy can be cured**	*Qualitative variable binary*<br>1=Yes<br>0=No	0.5	
**Knew how to treat leprosy**	*Qualitative variable binary*<br>1=On the way to the hospital<br>2=In the traditional	0.5	The correct answer has a weight of 0.5 and thus determines the score.
**Knew that leprosy can be treated in the hospital**	*Qualitative variable binary*<br>1=Yes<br>0=No	0.5	
**Knew leprosy treatment centres (CTAL)**	*Nominal qualitative variable (multiple choice with 1=Yes and 0=No for each modality)*<br>a) CTAL Davougon<br>b) CDTLUB Pobè<br>c) CTAL Dassa	0.75	Each correct answer has a weight of 0.25. Their sum determines the score
**Knew complications of leprosy in the absence of adequate treatment**	*Nominal qualitative variable (multiple choice with 1=Yes and 0=No for each modality)*<br>a) Claws<br>b) Bone resorption<br>c) Amputation d) Loss of sight<br>e) Plantar perforating ulcer	1.25	Each correct answer has a weight of 0.25. Their sum determines the score
