# Supplementary material for: Acceptability and feasibility of chemoprophylaxis with single-dose rifampicin in four leprosy-endemic districts in Benin
Source: PLoS Negl Trop Dis. 2025 Apr 28;19(4):e0013057. doi: 10.1371/journal.pntd.0013057 (PMC12058174; doi:10.1371/journal.pntd.0013057)
Supplement: S3 Appendix — (PDF) [file pntd.0013057.s003.pdf]

Questionnaire for contacts of leprosy patients for chemoprophylaxis

This questionnaire is designed to obtain the information necessary to assess your knowledge of leprosy and your acceptability of the preventive strategy of rifampicin chemoprophylaxis.

Thank you for your cooperation in advance.

Questionnaire number (not to be completed) /______/____/______/

SECTION 1: IDENTIFICATION AND SOCIO-DEMOGRAPHIC CHARACTERISTICS OF THE SURVEY

N°	Questions	Answers	Code	Jump
100	Date of Investigation	/______/_____/______/		
101	Department	Plateau<br>Zou	1<br>2	
102	Commune	Ketu<br>Djidja<br>Ouinhi<br>Zagnanado	1<br>2<br>3<br>4	
103	District	/______________________________/		
104	Village/Neighborhood	/______________________________/		
105	Surnames and first names			
106	Gender of the respondent<br>**Warning: Don't ask**	Masculine<br>Feminine	1<br>2	
107	How old are you now?	// in years gone by		
108	Marital status of the respondent	Bachelor<br>Married<br>Divorced<br>Separate<br>Widow/widower	1<br>2<br>3<br>4<br>5	
109	What is your socio-cultural group?	Fon<br>Mahi<br>Holly<br>Yoruba<br>Nago<br>Other<br>Specify______________	1<br>2<br>3<br>4<br>5<br>9	
110	What is your religion?	No Christian religion<br>Muslim<br>Traditional<br>Autre religion<br>Specify______________	1<br>2<br>3<br>4<br>9	
111	What level of education have you reached?	None<br>Primary<br>Secondary 1<br>Secondary 2<br>Upper	0<br>1<br>2<br>3<br>4	
112	What is your profession?	Merchant<br>Housewife<br>Student/Pupil<br>Official<br>Apprentice<br>Farmer<br>Fisherman<br>Artisan<br>Worker<br>Other	1<br>2<br>3<br>4<br>5<br>6<br>7<br>8<br>10<br>9	

N°	Questions	Answers	Code	Jump
		Specify_____________		

SECTION 2: KNOWLEDGE OF LEPROSY

200	What is leprosy called in your native language? ____________________ ____ ____			
201	How do you recognize a person with leprosy? *(multiple answers are possible)*		Yes No	
		(a) Visible deformation	1 0	
		b) Insensitive light spots	1 0	
		c) Gros nerfs	1 0	
		d) Don't know	1 0	
		(e) Other	1 0	
		Specify____________________________________ _____________		
202	What is/are the early sign (s) of the disease? *(multiple answers are possible)*		Yes No	
		(a) Visible deformation	1 0	
		b) Insensitive light spots	1 0	
		c) Gros nerfs	1 0	
		d) Don't know	1 0	
		(e) Other	1 0	
		Specify_____________________________		
203	What do you think is the cause of this disease? *(multiple answers are possible)*		Yes No	
		a) Bewitchment or bewitchment	1 0	
		b) Punition divine	1 0	
		(c) Natural Cause	1 0	
		(d) Micro-organism	1 0	
		e) Don't know	1 0	
		(f) Other	1 0	
		Specify____________________________________ _____________		
204	Can you get leprosy from another person?	Yes<br>Not	1<br>0	
205	How is leprosy transmitted? *(multiple answers are possible)*		Yes No	
		(a) From parent to child	1 0	
		(b) From a sick person to a healthy person	1 0	
		c) Does not transmit	1 0	
		d) Don't know	1 0	
		(e) Other	1 0	
		Specify_____________________________		
206	Can leprosy be cured?	Yes<br>Not	1<br>0	
207	How is this disease treated?	On the way to the hospital<br>In the traditional<br>Other<br>Specify____________	1<br>2<br>9	

N°	Questions	Answers	Code		Jump
208	In your opinion, can the disease be treated in a hospital?	Yes<br>No	1<br>0		If 0, change to 211
209	Where can I get treatment?	Health Center<br>CTAL<br>Other<br>Specify_____________	1<br>2<br>9		
210	What are some examples of a leprosy treatment centre (ETC) that you are familiar with.<br>*(multiple answers are possible)*		Yes	No	
		a) CTAL Davougon	1	0	
		b) CDTLUB Pobè	1	0	
		c) CTAL Dassa	1	0	
		(d) Other	1	0	
		Specify_____________________________			
211	What are the complications of leprosy if proper treatment is not provided?<br>*(multiple answers are possible)*		Yes	No	
		(a) Claws	1	0	
		b) Bone resorption	1	0	
		c) Amputation	1	0	
		(d) Perte de vue	1	0	
		e) Plantar perforating pain	1	0	
		(f) Other	1	0	
		Specify_____________________________			

SECTION 3: ACCEPTABILITY OF CHEMOPROPHYLAXIS

300	Will you accept medication to avoid getting leprosy?	Yes<br>No	1<br>0		If 1, go to 302
301	If not, why not?<br>Multiple Choice	I was away<br>I was sick<br>I was pregnant<br>I have doubts about the medication<br>The drug has side effects<br>I was not informed of the event<br>The doctors refused to give me the medicine<br>Other Reasons<br>Specify_____________	1<br>2<br>3<br>4<br>5<br>6<br>7<br><br>9		
302	If so, in what form(s) do you prefer to have these medications?<br>*(multiple answers are possible)*		Yes	No	
		(a) Tablet	1	0	
		(b) Syrup	1	0	
		c) Capsule	1	0	
		d) Injection	1	0	
		(e) Any form	1	0	
		f) Don't know	1	0	
		(g) Other	1	0	
		Specify_____________________________			

N°	Questions	Answers	Code	Jump
303	Will you be willing to travel a significant distance (> 5 km) to receive this drug?	Yes<br>Not	1<br>0	
304	Even if it takes you a whole day, will you accept this medication?	Yes<br>Not	1<br>0	
305	How often are you willing to take this medication?	Once<br>Twice<br>So many times<br>Don't know	1<br>2<br>3<br>4	
306	Will you still agree to take this medication even if you have noticed side effects (urine discoloration, digestive upset) when you first take it?	Yes<br>Not	1<br>0	
307	Are you willing to tell other people about this medication to encourage them to take it too?	Yes<br>Not	1<br>0	

Thank you for your participation.
