# Supplementary material for: Acceptability and feasibility of chemoprophylaxis with single-dose rifampicin in four leprosy-endemic districts in Benin
Source: PLoS Negl Trop Dis. 2025 Apr 28;19(4):e0013057. doi: 10.1371/journal.pntd.0013057 (PMC12058174; doi:10.1371/journal.pntd.0013057)
Supplement: S4 Appendix — (DOCX) [file pntd.0013057.s004.docx]

**Assessment the level of knowledge of PAL contact (Score)**

| **Level of knowledge (score)** | **Djidja**  **n (%)** | **Ouinhi**  **n (%)** | **Zagnanado**  **n (%)** | **Kétou**  **n (%)** | **Total**  **n (%)** |
| --- | --- | --- | --- | --- | --- |
| **[1-2[** | 5 (2,8) | 4 (5,0) | 1 (1,7) | 2 (2,7) | 12 (3,0) |
| **[2-3[** | 19 (10,6) | 7 (8,8) | 6 (10,0) | 17 (23,3) | 49 (23,9) |
| **[3-4[** | 69 (38,3) | 27 (33,8) | 17 (28,3) | 27 (37,0) | 140 (35,5) |
| **[4-5[** | 65 (36,1) | 32 (40,0) | 28 (46,7) | 24 (32,9) | 149 (37,8) |
| **= 5** | 12 (6,7) | 2 (2,5) | 4 (6,7) | 3 (4,1) | 21 (5,3) |
| **> 5** | 10 (5,6) | 8 (10,0) | 4 (6,7) | 1 (1,4) | 23 (5,8) |
| **Total** | 180 | 80 | 60 | 74 | 394 (100,0) |
